# Perioperative management of a pregnant patient with mediastinal tumor complicated by tuberculosis

**DOI:** 10.1186/s40981-017-0136-z

**Published:** 2017-12-16

**Authors:** Motoi Kumagai, Wakana Koishi, Hiroya Takahashi, Kenji Suzuki

**Affiliations:** Department of Anesthesiology, School of Medicine, Iwate Medical University, 19-1 Uchimaru, Morioka, 020-0023 Japan

**Keywords:** Cesarean section, Mediastinal tumor, Pregnancy, Tuberculosis

## Abstract

Mediastinal tumor in a pregnant woman, which had needed a multidisciplinary approach, was further complicated by tuberculosis. The clinical course of the current patient was very complicated. A 37-year-old female at 18 weeks of gestation with a mediastinal tumor was referred to our hospital due to dyspnea and orthopnea. The tumor compressed the left main bronchus causing bronchial stenosis. She was diagnosed with primary mediastinal large B-cell non-Hodgkin’s lymphoma. Delivery after 24 gestational weeks with ongoing chemotherapy was planned by a multidisciplinary team comprising obstetricians, anesthesiologists, neonatologists, and hematologists. Her symptoms improved with chemotherapy; however, she was later diagnosed with tuberculosis leading to chemotherapy interruption to treat tuberculosis. The following confirmation by negative sputum smear microscopy, an elective cesarean section with spinal anesthesia was performed at 33 weeks of gestation, and she safely delivered a female infant. At postoperative day 23, she died due to cardiopulmonary arrest, following an irreversible coma subsequent to brain metastasis of malignant lymphoma. The infant died of respiratory failure at postoperative day 18. This case illustrates several implications, such as the necessity of a thorough systemic examination and treatment approaches for mothers and neonates with suspected tuberculosis during the perioperative period, for considering similar cases with neoplasms.

## Background

Anterior mediastinal masses in pregnant patients are very rare and require a multidisciplinary approach for the diagnosis and treatment. We, herein, present a case that was further complicated by tuberculosis, requiring the cooperation of the entire hospital for proper management.

## Case presentation

**Fig. 1:**
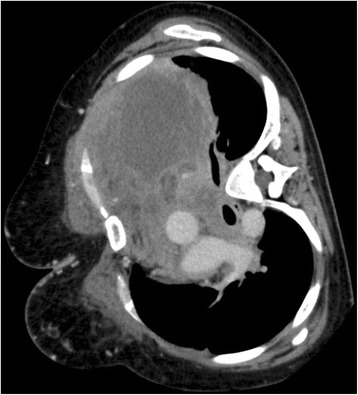
Computed tomography scan below the level of carina, which was taken on day 2 after admission, showing severe obstruction of the left main bronchus

A 37-year-old gravida 3 para 2 woman at 18 weeks of gestation with a mediastinal tumor was referred to our hospital due to dyspnea and orthopnea. She stated that her symptoms were intolerable and exaggerated in positions except over 60 degrees Fowler’s or orthopneic position. Although an irritating cough associated with mild dyspnea had been treated as bronchial asthma, her symptoms had not improved. Computed tomography (CT) showed a large mediastinal tumor measuring 18.0 × 15.5 cm, which occupied more than half the transthoracic diameter and compressed the left main bronchus, heart, and superior vena cava, causing bronchial stenosis and superior vena cava syndrome (Fig. 1). She had no clinically significant medical history except for situs inversus, no surgical or anesthetic history, and no known allergies. Left cervical lymph node biopsy led to a diagnosis of primary mediastinal large B-cell non-Hodgkin’s lymphoma (PMBCL). Chemotherapy with ongoing pregnancy was planned by hematologists due to the imminent threat of respiratory arrest and extremely preterm fetus. A multidisciplinary team comprising obstetricians, anesthesiologists, neonatologists, and hematologists, who convened once weekly, concluded that delivery after 24 gestational weeks with continuing chemotherapy was preferable. After 9 weeks of chemotherapy, the tumor in the left lung apex shrank, pneumatization was observed in the left lower lung field, and pericardial effusion was reduced. Oxygen flow rate was decreased from 4 to 1.5 L/min to maintain a SpO_2_ > 98%, and she was occasionally able to sleep in the supine position at night without dyspnea.

At 27 weeks of gestation, nodular shadows in the right hilar lesion were observed on a chest X-ray. Based on positive sputum smear microscopy and polymerase chain reaction (PCR), she was diagnosed with tuberculosis. Chemotherapy was interrupted to initiate tuberculosis treatment including isoniazid, rifapentine, and ethambutol. After 3 weeks of treatment, three consecutive samples of sputum smear microscopy were negative. Despite chemotherapy interruption, respiratory symptoms did not relapse.

Based on the multidisciplinary team’s decision, a cesarean section at 33 weeks of gestation was planned. It was decided that the infant should be treated as a tuberculosis patient after delivery, until confirmation of negative amniotic fluid by PCR analysis. Additionally, the operating room was temporarily remodeled as a negative-pressure room, and the air conditioning was turned off. As an anesthetic method, a combined spinal-epidural anesthesia (CSEA) was chosen, and the spinal block was specifically planned for lower abdomen below the T10 level where the surgical incision would be made, whereas epidural block was planned for below T5 level; percutaneous cardiopulmonary support was planned as standby.

All personnel wore N95 protective masks. An intra-arterial catheter was placed in the left radial artery. Baseline blood pressure, pulse, and pulse oximeter readings were 105/60 mmHg, 80 beats/min, and 99% on a nasal cannula with 4 L/min oxygen, respectively. An epidural catheter was inserted at the T11-T12 intervertebral space and threaded 5 cm cephalad, and spinal anesthesia was performed at the L2-L3 interspace. Hyperbaric 0.5% bupivacaine (11 mg) was administered into the subarachnoid space to obtain a block below the T5 level. Next, the cardiologist inserted a 14-F tube via the right femoral artery and a 17-F tube via the left femoral vein, with cardiopulmonary bypass pump readily accessible. The cesarean section was completed without complications. She did not experience any respiratory distress in the supine position during the operation and delivered a 1.6-kg female infant, with Apgar scores of 7 and 9 at 1 and 5 min, respectively. The operation time was 52 min, and total blood loss was 424 mL.

Starting on postoperative day 4, the patient developed nausea. Postoperative contrast-enhanced CT and magnetic resonance imaging showed brain metastasis of malignant lymphoma. The patient died of cardiopulmonary arrest following an irreversible comatose state on postoperative day 23. The infant was intubated due to respiratory failure from a groaning and retracted respiration and had a repeat pneumothorax. The neonatologist determined that the lung tissues were fragile, and she died of respiratory failure 18 days after birth.

## Discussion

She was approximately 5 months away from the expected delivery date. The physiological change progression during pregnancy, such as diaphragm elevation, could deteriorate the respiratory status. Chemotherapy continuation could harm the fetus and increase the tuberculosis relapse risk. Prolongation of pregnancy could increase the emergency cesarean section risk under insufficiently prepared conditions. Normal or induced labor with pushing or bearing down effort would increase maternal intra-abdominal and intrathoracic pressure, and prolonged labor might not be tolerated by severely dyspneic patients. Therefore, the elective cesarean section was planned for the time when her respiratory symptoms would disappear in the absence of tuberculosis treatment.

General anesthesia in patients with a mediastinal mass is reported to be associated with morbidity and mortality [1]. Spinal anesthesia can block the accessory respiratory muscles that might be critical for respiration in dyspneic patients [2]. The safety of epidural anesthesia in such cases has been reported [3, 4]; however, there remains a risk of analgesia failure, resulting in conversion to general anesthesia [5]. In the current case, spine caries was eliminated from the diagnosis, because she did not have back pain, stiffness, neurological abnormalities, and lumbago before and during admission [6]. Therefore, although CSEA had been planned, it was altered based on the patient’s respiratory status. Extracorporeal circulation is reported to be necessary in cases where intubation or positive-pressure ventilation was predicted to be difficult [7, 8]. Although the situs inversus could make the cannulation difficult, it was performed without difficulty by the cardiologists using portable ultrasound and X-ray.

This case illustrates several implications. Brain metastasis of PMBCL is rare [9]. The preoperative systemic examination is suggested in patients with neoplasms. The lung tissue fragility of the infant might be caused by chemotherapy during the prenatal period [10]. Due to an efficient infection control, none of the medical personnel was infected with tuberculosis. The infant temperature was close to the lower limit (35.7 °C at the skin surface and 36.5 °C at the rectum). A heating appliance such as a far-infrared radiation heater that did not cause airflow could have been employed [11].

## Conclusion

We experienced the perioperative management of a pregnant patient with mediastinal tumor complicated by tuberculosis that requires the cooperation of the entire hospital for proper management, including not only the multidisciplinary approach but also the preparation of medical devices, alterations in the operating room environment, and the perioperative systemic examination taking the metastasis of neoplasms into consideration.
